# Hypernatremia is associated with mortality in severe elderly sepsis patients

**DOI:** 10.1371/journal.pone.0310245

**Published:** 2024-11-08

**Authors:** Xu Liu, Yalin Hong, Bingchen Li, You Xu, Nianci Wang, Han Liu, Ying Liu

**Affiliations:** 1 Department of Critical Care Medicine, Nanjing First Hospital, Nanjing Medical University, Nanjing, China; 2 Department of Critical Care Medicine, Jiangsu Provincial Government Hospital, Nanjing Medical University, Nanjing, China; 3 Department of Emergency Medicine, Nanjing First Hospital, Nanjing Medical University, Nanjing, China; Universita degli Studi di Parma, ITALY

## Abstract

**Objective:**

To explore the relationship between hypernatremia and 28-day mortality in elderly sepsis patients.

**Methods:**

A total of 179 elderly patients (age ≥65 years) with elevated serum sodium admitted to the Department of Critical Care Medicine of Nanjing Hospital affiliated with Nanjing Medical University from September 2021 to September 2022 were included in this retrospective observational study. The clinical data of all patients were collected, and the patients were divided into septic group and nonseptic groups according to the Sepsis 3.0 definition. The clinical features, acute physiological and chronic health II score (APACHE II score), mechanical ventilation time, serum sodium value and duration of serum sodium elevation were compared between the two groups. ROC curves were drawn to evaluate the predictive value of each index on the prognosis of sepsis patients, and Kaplan‒Meier survival analysis was carried out on patients with different serum sodium peaks.

**Results:**

(1) The changes in serum sodium within 48 hours after admission in the sepsis group were small and statistically significant compared with those in the nonsepsis group (*P* = 0.039); however, the serum sodium elevation duration was longer (*P* = 0.018). (2) Compared with nonseptic patients, the 7-day mortality of septic patients was higher (15.8 vs. 7.7, *P*<0.001). The 28-day mortality of septic patients was higher than that of nonseptic patients, but there was no significant difference between the two groups (*P* = 0.086). (3) The serum sodium level in the sepsis group was higher than that in the nonsepsis group on the 1st, 3rd, 5th and 7th days (*P*<0.001). There was no significant difference in mechanical ventilation time or duration of stay in the ICU between the two groups. (4) The ROC curve analysis showed that the peak value of serum sodium had predictive value for the prognosis severity of elderly patients with sepsis. The area under the curve (AUC) was 0.753, the 95% confidence interval (95% CI) was 0.639~0.867, and the best cut-off value was 154.9 mmol/L. (5) According to the best cut-off value of the serum sodium peak, the septic patients were divided into two groups: the peak value of serum sodium was ≥154.9 mmol/L (group A), and the peak value of serum sodium was <154.9 mmol/L (group B). Among them, the case fatality rate was higher at 7 days and 28 days when the peak value of serum sodium was ≥154.9 mmol/L (group A) (22.0% vs. 8.6%); the χ^2^ value was 35.379, *P*<0.05; 75.6% vs. 37.1%, χ^2^ = 14.21, *P* = 0.003). There was no significant difference in mechanical ventilation time or duration of stay in the ICU between the two groups. (6) Kaplan‒Meier survival analysis showed that the median survival time of patients with a serum sodium peak ≥154.9 mmol/L (group A) was significantly shorter than that of patients with a serum sodium peak < 154.9 mmol/L (group B) (16.7±1.4 d vs. 24.8±1.2 d, *P* <0.05).

**Conclusions:**

The serum sodium increase in elderly sepsis patients lasts for a long time, and the serum sodium fluctuation is relatively small. The serum sodium peak value has predictive value for 28-day mortality.

## Introduction

Hypernatremia is defined as a serum sodium concentration exceeding 145 mmol/L, and the incidence rate in severe patients can reach 10% [1,2]. In elderly patients, the occurrence of hypernatremia is related to the imbalance of blood volume, the age-dependent degeneration of osmotic pressure receptors in the brain stem, the weakening of regulation, the trauma of cerebral haemorrhage, parenteral nutrition feeding and kidney diseases [3,4]. Hypernatremia is a pathophysiological process of water and electrolyte imbalance; the harm it causes cannot be ignored; and it can cause functional damage to the nervous system, immune system and endocrine system [4,5]. Previous studies have found that in burn patients, hypernatremia leads to progressive deepening and necrosis of the wound, prolongs the hospitalization time of patients and leads to poor prognosis [6]. In patients with heart failure, abnormal serum sodium is an independent predictor of death [7], and a study of COVID-19 patients also found that hypernatremia is related to poor prognosis [8].

Sepsis is a life-threatening organ dysfunction that is caused by the host’s maladjustment in response to infection. Elderly critically ill patients have many basic diseases and relatively low immune function, and the probability of sepsis is higher in these patients [9,10]. With the ageing of the population, the disease burden of sepsis increases, so it is very important to study the predictive factors of death in sepsis patients.

In summary, the clinical prognosis of patients with sepsis and hypernatremia is poor, and there are few studies on the clinical outcome of sepsis complicated with hypernatremia in national and international literature. Through a retrospective analysis of senile sepsis patients complicated with hypernatremia, this paper aims to explore whether hypernatremia in elderly sepsis patients is independently related to their prognosis.

## Materials and methods

### Research subjects

A total of 179 patients with hypernatremia residing in the Department of Critical Care Medicine of Nanjing Hospital Affiliated to Nanjing Medical University from September 2021 to September 2022 were included.

### Inclusion criteria

**(1)** Age ≥65 years old; (2) duration of stay in the ICU >4 days; (3) serum sodium > 145 mmol/L within 48 hours after admission to the ICU

### (2) Exclusion criteria

(1) Age < 65 years old; (2) duration of stay in the ICU less than 4 days; (3) diabetic ketoacidosis, primary aldosteronism, Cushing’s syndrome, and the use of drugs affecting serum sodium.(sodium preserving and potassium excreting drugs,as fruosemide).

### Study groups

According to the definition of Sepsis 3.0 [11], patients were divided into a septic group and a nonseptic group, and ROC curves were drawn to evaluate the predictive value of each index for the prognosis of sepsis patients.

### Clinical data

In this study, a retrospective observational study design was adopted to collect basic clinical information of patients, including sex, age, values of any serum sodium elevations on the 1st, 3rd, 5th, and 7th days, and the peak serum sodium; to calculate the maximum change value within 48 hours after admission; and to record the duration of the patient’s stay in the ICU, mechanical ventilation time, APACHE II score, the expected risk of death is based on a software scoring system composed of acute physiological score, age, chronic disease, and risk coefficient, reflecting the severity of the disease,and other indicators. The primary study endpoints were the 7-day and 28-day mortality rates, while the secondary study endpoints were the duration of stay in the ICU and mechanical ventilation time.

### Statistical analysis

SPSS 25 statistical software was used to analyse the data, and GraphPad Prism 9 was used for graphing. To compare numerical variables, the Kolmogorov‒Smirnov normality test and Levene homogeneity of variance test were performed. Measurement data that conformed to a normal distribution are expressed as the mean ± standard deviation (±s) and were compared using the independent-samples t test. Measurement data that did not conform to a normal distribution are expressed as the median (quartile spread) [M (QL, QU)] and were compared using the Mann‒Whitney U test. Count data are expressed as the number of patients and were compared using the *χ*^*2*^ test. ROC curves were plotted to further analyse the predictive value of elevated serum sodium on mortality in elderly sepsis patients. Taking α = 0.05, *P*<0.05 was considered statistically significant; all tests were two-sided.

## Results

### Comparison of general patient data

A total of 179 patients were included in this study, and they were divided into a sepsis group and a nonsepsis group according to whether they had sepsis. There were 76 patients in the sepsis group and 103 patients in the nonsepsis group. In the sepsis group, there were 51 males and 25 females, aged 78.6±8.7 years old. In the nonsepsis group, there were 68 males and 35 females, aged 77.6±9.7 years old. There was no significant difference in the average age and sex of the patients between the two groups. The two groups included 39 patients (51.3%) and 53 patients (51.4%) with hypertension, 17 patients (22.3%) and 20 patients (19.4%) with diabetes, 33 patients (43.3%) and 22 patients (21.3%) with kidney disease, 24 patients (31.5%) and 36 patients (35.0%) with coronary heart disease, respectively. There were 24 patients (31.5%) and 39 patients (37.8%) with cerebrovascular diseases in each group, respectively. The proportion of patients with sepsis combined with basic diseases, such as diabetes and kidney disease, was higher than that of the nonsepsis group, and the proportion of coronary heart disease and cerebrovascular disease in the nonsepsis group was higher, however there was no significant difference. There was no significant difference in the APACHE II score, estimated risk of death, duration of stay in the ICU, or mechanical ventilation time between the two groups of patients (Table 1).

**Table 1:** Basic characteristics of patients with elevated serum sodium.

Item	Sepsis (n = 76)	Non-sepsis (n = 103)	*χ*^*2*^ */ t / F/Z value*	*P value*
Age (years)	78.6±8.7	77.6±9.7	0.512	0.512
Sex (male, %)	51(67.1)	68(66.1)	0.023	0.87
APACHEII score	25.9±7.2	26.9±8.8	2.598	0.109
Expected risk of death (%)	48.6±18.0	46.2±20.1	2.831	0.094
Mechanical ventilation time (d)	12.5±11.0	11.8±15.7	0.170	0.681
Duration of stay in ICU (d)	18.3±13.1	17.9±17.1	-1.383	0.167
Chronic Disease				
Hypertension (%)	39(51.3)	53(51.4)	0.140	0.709
Diabetes (%)	17(22.3)	20(19.4)	0.232	0.63
Kidney disease (%)	33(43.3)	22(21.3)	10	0.002*
Coronary heart disease (%)	24(31.5)	36(35.0)	0.223	0.63
Cerebrovascular disease (%)	24 (31.5)	39 (37.8)	0.757	0.38

APACHE II, Acute Physiology and Chronic Health Evaluation

*P<0.05

**P<0.001.

### Relationship between serum sodium levels and prognosis in elderly patients

The changes in serum sodium levels between septic patients and nonseptic patients were analysed. Compared with nonseptic patients, the changes in serum sodium in septic patients at 48 hours were small and statistically significant (*P* = 0.039), and the duration of serum sodium increase was longer (*P* = 0.018). The serum sodium levels of septic patients and nonseptic patients were significantly different on the first day, the third day, the fifth day, and the seventh day, and the peak value of serum sodium was also significantly different (*P* < 0.008). The 7-day mortality and 28-day mortality were calculated with 7 days and 28 days, respectively, as the observation end points. The results showed that the 7-day mortality in the sepsis group was higher than that in the nonsepsis group (15.8% vs. 7.7% *P* < 0.001), and although the 28-day mortality in the sepsis group was higher than that in the nonsepsis group, there was no significant difference (*P* = 0.086) (Table 2).

**Table 2:** Relationship between serum sodium and case fatality rate in patients with sepsis and non-sepsis.

Item	Sepsis (n = 76)	Non-sepsis (n = 103)	*χ*^*2*^ */ t / F/Z value*	*P value*
Serum sodium D1 (mmol/L)	148.7±1.7	146.9±1.4	-73.48	<0.001**
Serum sodium D3 (mmol/L)	151.4±3.3	150.6±2.7	-15.98	<0.001**
Serum sodium D5 (mmol/L)	153.7±6.1	149.9±1.0	-56.32	<0.001**
Serum sodium D7 (mmol/L)	152.9±4.1	148.7±1.4	-97.75	<0.001**
Serum sodium peak (mmol/L)	156.2±4.5	151.6±2.7	-7.316	<0.001**
48h changes in serum sodium (mmol/L)	6.4±3.6	7.8±4.7	4.316	0.039*
Duration of serum sodium elevation (d)	8.1±3.8	7.0±3.7	-2.364	0.018*
7-day mortality (%)	15.8	7.7	107.939	<0.001**
28-day mortality (%)	57.8	55.3	2.955	0.086

Serum sodium D1: The first day value of serum sodium; Serum sodium D3: The value of serum sodium on the 3rd day; Serum sodium D5: The value of serum sodium on the 5th day; Serum sodium D7: The value of serum sodium on the 7th day; Serum sodium peak: Within 7 days, serum sodium peak; 48h changes in serum sodium: refersto the maximum change value within 48 hours after admission

* *P*<0.05

* * *P*<0.001.

### ROC curve plotting for predicting the risk of death in elderly patients with sepsis and hypernatremia

**Fig 1:**
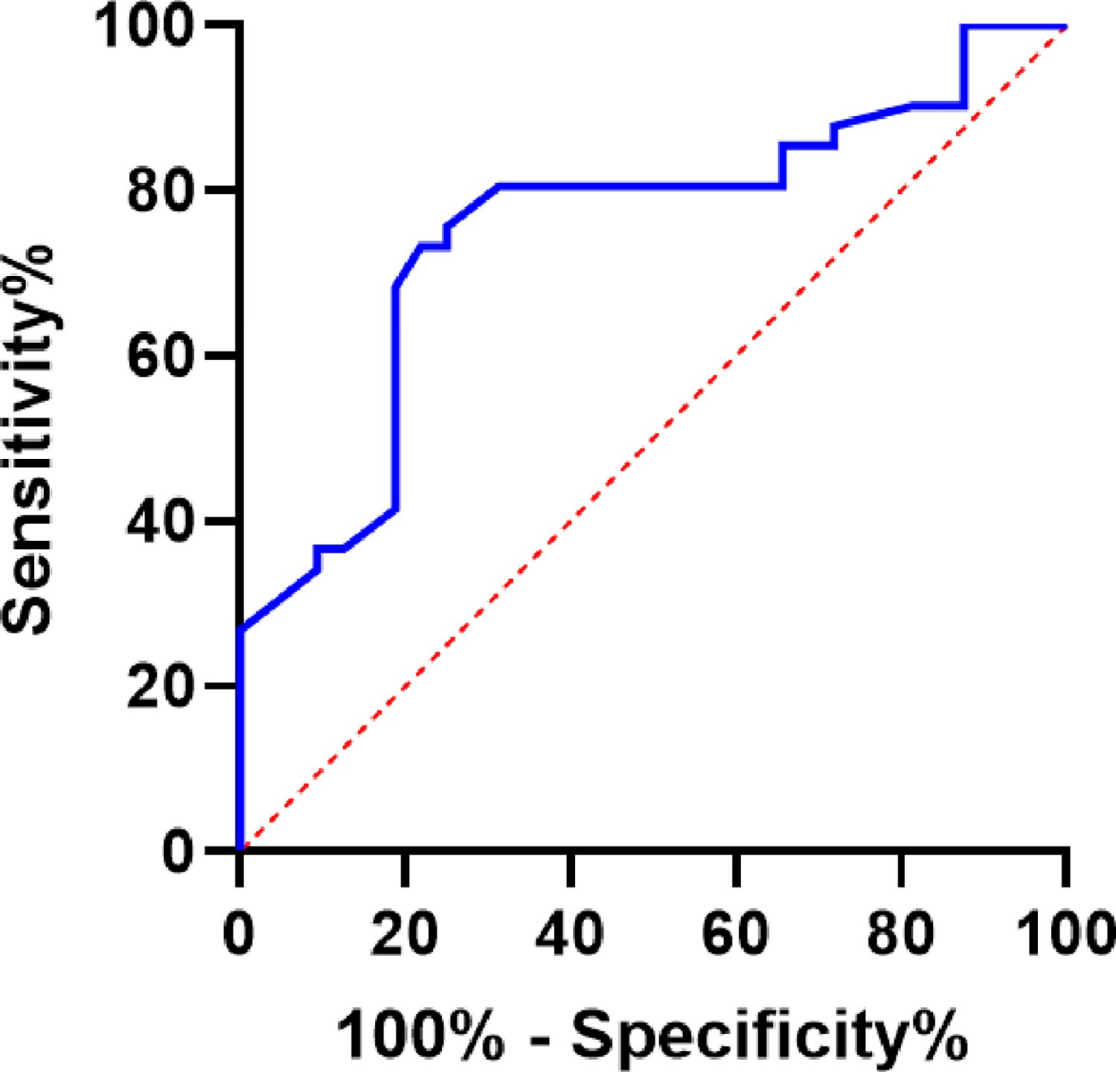
ROC curve of serum sodium peak on 28-day prognosis in elderly sepsis patients.

ROC curve analysis was used to predict the death risk of elderly patients with elevated serum sodium complicated with sepsis. The results showed that the areas under the Serum sodium D1,48h changes in serum sodium and Duration of serum sodium elevation ROC curve were 0.587, 0.602 and 0.517, respectively, while the area under the peak curve of serum sodium was 0.753, *P*<0.05. It is suggested that the peak value of serum sodium can predict the 28-day death of elderly patients with sepsis and hypernatremia. The 95% confidence interval (95% CI) was 0.639~0.867, and the best cut-off value was 154.9 mmol/L, with a corresponding sensitivity of 73.2%, a specificity of 78.1% and a Youden index of 0.51 (Table 3, Fig 1).

**Table 3:** Area indicators under serum sodium peak curve.

Item	AUC	95%CI	*P value*	Sensitivity (%)	specificity (%)	Youden index	Cut-off value
Serum sodium peak	0.753	0.639~0.867	<0.001	73.2	78.1	0.51	154.9

### The predictive value of serum sodium levels at different levels for the prognosis of elderly sepsis patients

To further analyse the predictive value of the serum sodium peak value for patient prognosis, the patients with sepsis were regrouped according to the best cut-off value of the serum sodium peak value and were divided into two groups: the A group with a serum sodium peak value ≥154.9 mmol/L and the B group with a serum sodium peak value < 154.9 mmol/L. The results showed that there were 41 patients in group A and 35 patients in group B. The mortality of the patients in group A at 7 days and 28 days was significantly higher than that in group B (*P*<0.05), but there was no significant difference in the duration of stay in the ICU or mechanical ventilation time between the two groups (Table 4).

**Table 4:** Comparison of adverse prognosis in patients with different levels of serum sodium peak.

Item	A (≥154.9, n = 41)	B (<154.9, n = 35)	*χ*^*2*^ */ t / F/Z value*	*P value*
Duration of stay in ICU (d)	18.2±21.3	18.5±10.6	-1.226	0.220
Mechanical ventilation time (d)	13.6±21.5	13.5±10.8	-0.763	0.446
7-day mortality rate (%)	22.0	8.6	35.379	<0.001**
28-day mortality rate (%)	75.6	37.1	14.21	0.003*

* *P*<0.05

* * *P*<0.001.

### Survival analysis of patients with different levels of serum sodium peak

**Fig 2:**
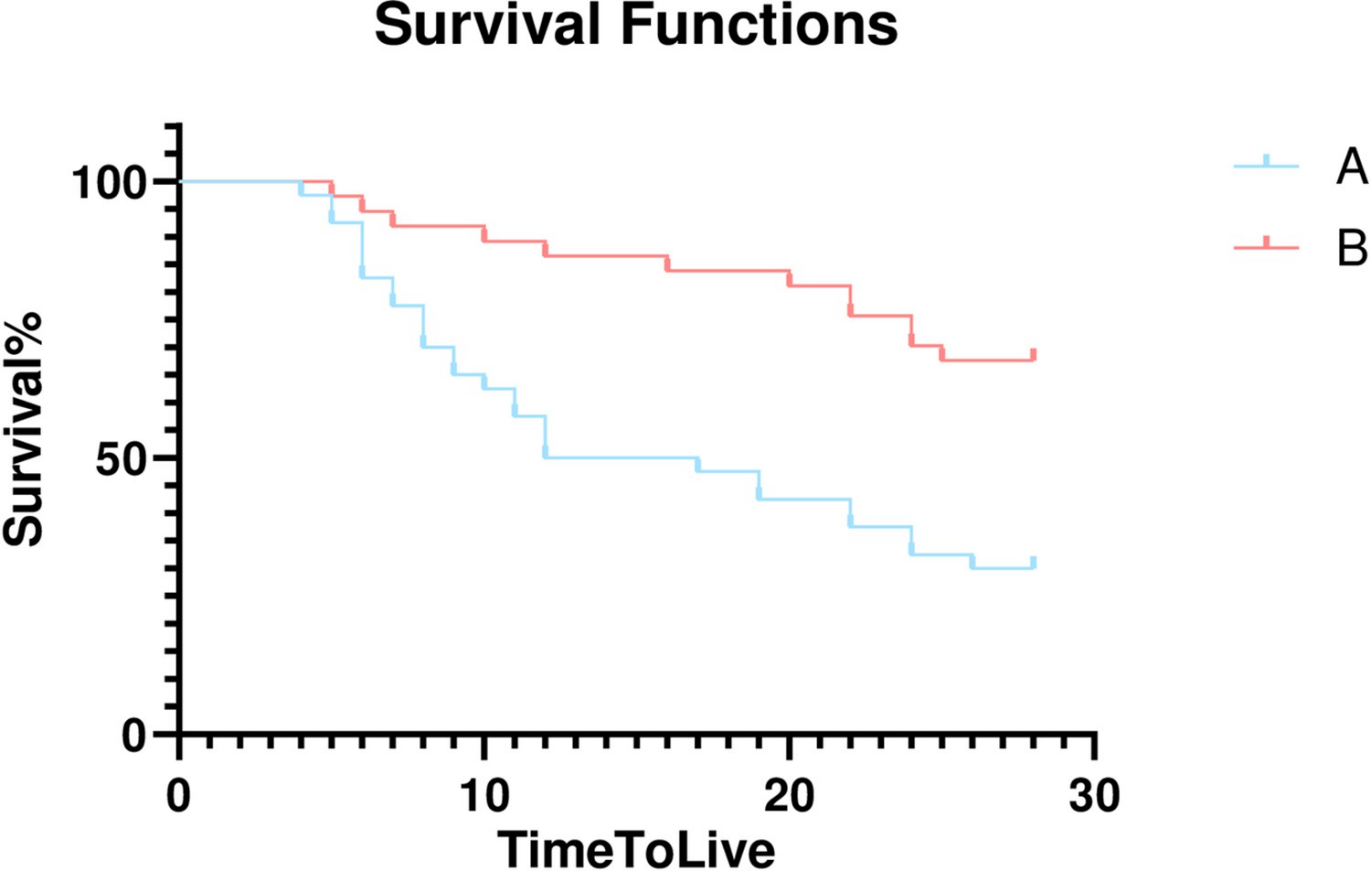
KM curves of different levels of serum sodium peak on the prognosis of elderly patients with sepsis and hypernatremia. Group A had a serum sodium peak of ≥ 154.9mmol/L, while Group B had a serum sodium peak of<154.9mmol/L in elderly patients with sepsis and hypernatremia.

By the end of the study, Kaplan‒Meier survival analysis curves were drawn for patients with serum sodium peak values greater than or equal to 154.9 mmol/L (group A) and those with peak values less than 154.9 mmol/L (group B). The results showed that the median survival time of group A was 16.7±1.4 days, while that of group B was 24.8±1.2 days, suggesting that the survival time of group B was significantly higher than that of group A (*P*<0.001) (Fig 2).

## Discussion

In this retrospective study of elderly patients in the ICU, our goal was to evaluate the influence of elevated serum sodium on the in-hospital mortality of elderly patients with sepsis in the ICU. Within 24 hours after admission to the ICU, the peak serum sodium level is related to the risk of death in elderly patients with sepsis and hypernatremia. Sepsis is a common life-threatening disease in the ICU and has a high incidence, but the basic immunity of the elderly is poor [12]. In this study, elderly patients with septic hypernatremia had a worse prognosis and a higher 7-day mortality than elderly patients without septic hypernatremia. Studies have shown that more than 70% of patients with severe sepsis complicated with hypernatremia die three days after onset [13], which is consistent with the results of this study, suggesting that the short-term mortality rate is high. There was no significant difference in the APACHE II scores between the two groups in this study, suggesting that the basic illness severity of the two groups was similar, indicating that hypernatremia is more likely to be accompanied by poor prognosis in elderly sepsis patients.

By analysing the serum sodium levels of the two groups, we found that the changes in serum sodium in the sepsis group were small within 48 hours after admission, and the duration of the increase was longer. Because serum sodium mainly depends on central receptors and renal regulation [4], the proportion of patients with renal diseases in the sepsis group was higher in this study, and the ability of patients to self-regulate their serum sodium was poor. According to the guidelines for the management of sepsis haemodynamics [14], patients with septic shock are mostly given sodium-containing balanced fluids for fluid resuscitation after admission, and the use of this treatment can be explained by the synergistic effect of the above factors. In contrast to previous studies, this study shows that the maximum change value of elderly patients with sepsis is small within 48 hours, while previous studies have shown that the greater the difference between the maximum and minimum values of serum sodium during hospitalization is, the higher the mortality rate, and the difference in serum sodium can be used to predict the mortality rate in hospitals [15–19]. However, the above research groups have not conducted subgroup studies, and there is population heterogeneity. This study is different from previous studies; the research subjects were elderly patients; and the population homogeneity was high. The fluctuation of the serum sodium in these patients is small, but its peak duration is longer.

Previous studies have shown that the increase in serum sodium in patients with spontaneous cerebral haemorrhage is related to their prognosis [20]. In this study, the subgroup analysis of elderly patients with sepsis showed that the peak value of serum sodium can predict the 28-day mortality of patients, and the peak value of serum sodium > 154.9 mmol/L is related to 28-day mortality, which is consistent with previous research results. According to the subgroup analysis of sepsis patients with the best cut-off value, patients with a serum sodium peak value greater than or equal to 154.9 mmol/L had higher mortality at 7 days and 28 days, and the 28-day survival analysis also showed that the median survival time was shorter, suggesting that patients with a serum sodium peak value greater than 154.9 mmol/L had a worse prognosis. Elderly patients with sepsis have a high mortality rate, and when they are complicated with hypernatremia, they may develop mental changes or even become comatose due to brain cell dehydration [21]. The decrease in urine volume leads to an increase in renal burden, which leads to acute renal injury [22], thus causing a secondary blow to patients and leading to an increase in hospitalization mortality. Therefore, in the treatment of sepsis patients, it is necessary to strengthen the monitoring of serum sodium levels in elderly sepsis patients and intervene as soon as possible, especially to prevent iatrogenic increases in serum sodium, to improve the hospitalization outcome of patients.

The advantage of this study is its subgroup analysis, aiming at the specific population of elderly sepsis complicated with high serum sodium as the research object with good homogeneity. However, there are some limitations in this study. First, the research population was single, and the research subjects were elderly patients, which is a not universal population. Moreover, the sample size of the sepsis group was small, which may cause bias in the results. Second, the specific cause of hypernatremia was not analysed, and the influence of hormones regulating serum sodium levels was not ruled out. Third, there was no stratified study on serum sodium levels. Fourth, because this study was an observational study, the causal relationship cannot be inferred. Although age and sex were controlled for, there may still be other complicated factors that affect the research results.

## Conclusion

The increase in serum sodium in elderly patients with sepsis lasts for a long time, and the peak value of serum sodium is related to the 28-day mortality rate. Therefore, it is necessary to strengthen the monitoring of serum sodium levels in elderly patients with sepsis and intervene as soon as possible to improve the hospitalization outcome of patients.
